# Racial variation in the number of spontaneous abortions before a first successful pregnancy, and effects on subsequent pregnancies

**DOI:** 10.1016/j.ijgo.2015.01.004

**Published:** 2015-06

**Authors:** Clare T. Oliver-Williams, Philip J. Steer

**Affiliations:** a Department of Public Health and Primary Care, University of Cambridge, Cambridge, UK; b Academic Department of Obstetrics and Gynaecology, Imperial College London, Chelsea and Westminster Hospital, London, UK

**Keywords:** Preterm birth, Race, Racial group, Small for gestational age, Spontaneous abortion

## Abstract

**Objective:**

To assess the relationship between race and spontaneous abortion, whether the relationship varies by risk factors, and the effect of spontaneous abortions on subsequent pregnancies.

**Methods:**

A retrospective study was undertaken using data collected in London, UK, between 1988 and 2000. Logistic regression evaluated histories of spontaneous abortion and associations with small-for-gestational-age and preterm births in black African, black Caribbean, and South Asian women relative to white European women. Interactions with risk factors were assessed.

**Results:**

Overall, 196 040 women were included. Compared with white Europeans, the odds of a previous spontaneous abortion were increased in black African (adjusted odds ratio [aOR] 1.20; 95% confidence interval [CI] 1.12–1.29) and black Caribbean women (aOR 1.31; 95% CI 1.21–1.41). The strength of the association with black African race increased with age (*P* = 0.002), and the association with South Asian race increased with age and body mass index (*P* < 0.001 for both). Spontaneous abortion was associated with preterm birth in all races, but was strongest in black African women (aOR 1.47; 95% CI 1.29–1.67).

**Conclusion:**

The greater incidence of spontaneous abortion in black African and black Caribbean women should prompt further study of risk factors in relation to race. The interaction with age in black African and South Asian women could be important for counseling in relation to timing of pregnancy.

## 1 Introduction

Spontaneous abortion is a frequent adverse pregnancy outcome, occurring in approximately 12%–24% of clinically recognized pregnancies [1,2]. However, few studies have assessed racial/ethnic variation in incidence of spontaneous abortion. Two studies found an increased incidence in Afro-Caribbean women, although the increase ranged from 50% [3] to 300% [4]. Additionally, neither study assessed whether the increased incidence was modified by known risk factors, such as maternal age and body mass index (BMI).

Women who have experienced spontaneous abortion have increased rates of adverse outcomes in their next continuing pregnancy, such as preterm birth and small-for-gestational-age neonates [5]. Moreover, black and South Asian women are at an increased risk of preterm birth [6,7] and small-for-gestational-age neonates [6], irrespective of any previous spontaneous abortions. The risk in these women could be a result of genetic differences between ethnic/racial groups, because differences are found even after correction for socioeconomic risk factors [7], although residual lifestyle and cultural factors could remain after such adjustment. Nevertheless, these differences could have implications for other pregnancy outcomes, including spontaneous abortion. However, whether the effect of previous spontaneous abortion on these outcomes varies between ethnic/racial groups does not seem to have been reported previously. Given the greater risk of neonatal death and disability arising from these outcomes, and the increasing rates of preterm birth in most countries with reliable data [8], it is important to identify women at risk of adverse pregnancy outcomes.

Therefore, the aim of the present study was to determine: 1) whether the reported history of spontaneous abortion varied by race among nulliparous women; 2) whether risk factors such as BMI and maternal age had a differential effect between races on incidence of spontaneous abortion; and 3) the effect of spontaneous abortion on subsequent preterm birth and small-for-gestational-age neonates, by racial group. Race refers to genetic ancestry and reflects physical differences that have evolved in geographically isolated groups over millennia, whereas ethnic origin refers to social traits such as culture and diet. The two are not always the same (people can change their culture but not their genetics). In the present study, the descriptor “race” will be used.

## 2 Materials and methods

A retrospective study was undertaken using data from the St Mary’s Maternity Information System, which contains information about 585 291 women who attended 15 maternity units in the North West Thames Region of London, UK, for prenatal care and delivery between 1988 and 2000. In 2002, ethical approval was obtained from St Mary’s local research ethics committee for multiple uses of the data. Because data had been routinely collected and anonymized, patient consent was not required.

The North West Thames Region had a population of 3.3 million in 1988 and 3.8 million in 2000, as well as various racial groups, and suburban, rural and metropolitan areas [9,10]. Information was collected on demographic characteristics, medical history, and outcomes of all women and newborns from the first prenatal visit until 28 days after delivery. Data were entered into the system by trained clerks or midwives with online validation and standardized clinical definitions.

Only nulliparous women were included within the present analysis to exclude any bias that could be caused by including women more than once and to eliminate the effect of parity. Consequently 332 302 pregnancies were excluded. Additionally, inclusion was restricted to women aged 13–45 years at delivery who had a BMI (calculated as weight in kilograms divided by the square of height in meters) of 15–40 and delivered a baby with a birth weight greater than 500 g between 24 and 43 weeks of pregnancy. These criteria lead to the exclusion of 32 523 women. Additionally, 24 426 women were excluded because information about race was missing or because they were of mixed race or from racial groups with fewer than 15 000 women in the dataset. In analyses of subsequent outcomes, women with missing gestational age or birth weight were excluded.

Race was self-reported. White European, black African, black Caribbean, and South Asian racial groups were chosen for the present analysis because most participants in the dataset belonged to one of these groups. The South Asian group included women from India, Pakistan, and Bangladesh.

Previous spontaneous abortions were self-reported (0, 1, 2, or ≥ 3) and assessed as a continuous variable. The comparison group was no previous spontaneous abortions. Gestational age and birth weight were recorded at delivery. Preterm birth was defined as delivery before 37 completed weeks of gestation. Gestational age was calculated from the first day of the last menstrual period or measurements taken from fetal ultrasonography before 24 weeks of gestation if the last menstrual period was uncertain or cycle length variable [11]. Small-for-gestational-age birth weight, defined as a weight below the 10th centile corrected for sex and gestational age at birth, was calculated using birth weight standards for nulliparous English women [12].

Marital status (married, unmarried with a partner, or unmarried without a partner), cigarette smoking (current or non-smoker), and histories of induced abortion, hypertension, renal disease, cardiac disease, and epilepsy were self-reported. Diabetes status was reported by midwives at delivery and classified as established (pregestational) diabetes, gestational diabetes (diagnosed for the first time in the index pregnancy), or no diabetes. Women recorded as not having diabetes did not report the disorder and were not on a calorie-restrictive diet, oral hypoglycemics, or insulin.

Maternal age was defined as the mother’s age on the day of her child’s birth. BMI was assessed at the first prenatal visit, using either self-reported or measured weight. Socioeconomic deprivation was evaluated using the Carstairs socioeconomic deprivation score [13], which is based on census data on car ownership, unemployment, overcrowding, and social class within postcode sectors of residence containing approximately 16 000 residents. Five categories were constructed (1 = least deprived, 5 = most deprived).

Missing data was assumed to be missing at random and was dealt with using multiple imputation by chained equations [14]. Imputation was used for marital, smoking, and diabetes statuses; Carstairs deprivation score; BMI; and histories of hypertension, renal disease, cardiac disease, and epilepsy. All variables except for BMI had less than 10% of data missing; BMI had 17.8% of data missing. Five imputations were created using a set of appropriate imputation models constructed of all covariates and outcome variables, stratified by race. To reflect the proportion of missing data in BMI, an additional 15 imputations were created to verify that the results were robust to the number of imputations used.

Non-imputed data were summarized by race. Continuous variables were summarized by median and interquartile range, and compared across racial groups using Kruskal-Wallis tests. Categorical variables were summarized by number and percentage, and groups were compared using χ^2^ tests.

For imputed data, logistic regression was used to calculate odds ratios (ORs) and 95% confidence intervals (CIs) for previous spontaneous abortions within each racial group and for the whole population. The three minority races (black African, black Caribbean, and South Asian) were compared with white Europeans to maintain consistency with most literature on racial disparities. Effect modification was evaluated through inclusion of an interaction term between race and the potential effect modifier. Interactions were assessed using the Wald test.

Next, logistic regression was used to quantify the association between spontaneous abortion and both preterm birth and low birth weight in the next continuing pregnancy, using imputed data. Dose–response relationships were evaluated by including the exposure as a continuous variable in the model and evaluating the Wald test.

*P* values were two-sided, and *P* < 0.05 was considered statistically significant. A Bonferroni correction was performed when multiple comparisons were made. Statistical analyses were performed using Stata version 12.1 (StataCorp, College Station, TX, USA).

## 3 Results

A total of 196 040 nulliparous women were included in the present analysis. Black Caribbean women comprised the smallest group (3.0% of the sample), whereas white European women was the largest (80.9%). All characteristics varied by race, including diabetes status, BMI, maternal age, and number of previous spontaneous abortions (*P* < 0.001 for all) (Table 1). The group with the highest proportion of women who had had a previous spontaneous abortion was black Caribbean (15.7%); the group with the lowest proportion was South Asian women (11.8%).

The unadjusted logistic regression analysis showed that, compared with white European women, the odds of a previous spontaneous abortion were increased in black African women (OR 1.10; 95% CI 1.03–1.18) and black Caribbean women (OR 1.16; 95% CI 1.08–1.25), but reduced among South Asian women (OR 0.83; 95% CI 0.80–0.87).

Table 2 shows the factors associated with previous spontaneous abortion for the whole population and each race, after adjustment for potential confounders. Black African and black Caribbean races were independently and positively associated with spontaneous abortion, whereas South Asian women had marginally reduced odds of previous spontaneous abortion.

Maternal age at delivery was a positive independent risk factor for all races (Table 2). Within each racial group, cigarette smoking, established diabetes, or subsequent development of gestational diabetes (indicating a pre-diabetic state before pregnancy) were the factors most strongly associated with previous spontaneous abortions. Thus, these three factors were also most strongly associated with spontaneous abortion within the population as a whole.

When interactions between risk factors and race were included in the multivariate logistic regression model, BMI (*P* < 0.001) and maternal age at delivery (*P* < 0.001) were found to be positive effect modifiers for South Asian women: the strength of the association between previous spontaneous abortion and South Asian women increased with increasing BMI and age relative to white European women. Similarly, a positive interaction with age was found for black African women (*P* = 0.002).

Comparisons by age group showed that, relative to white European women aged 21–25 years, South Asian women aged younger than 20 years or 21–25 years were less likely to have had a previous spontaneous abortion (Table 3). This difference disappeared in older age groups. Odds were also reduced in black African and black Caribbean women aged younger than 20 years. However, odds were increased in all women aged 26 years or older, irrespective of racial group.

Comparisons by BMI showed that the odds of spontaneous abortion relative to white European women with a normal BMI were greatest for overweight/obese black African, and lowest for normal and underweight South Asian women (Table 3).

**Fig. 1:**
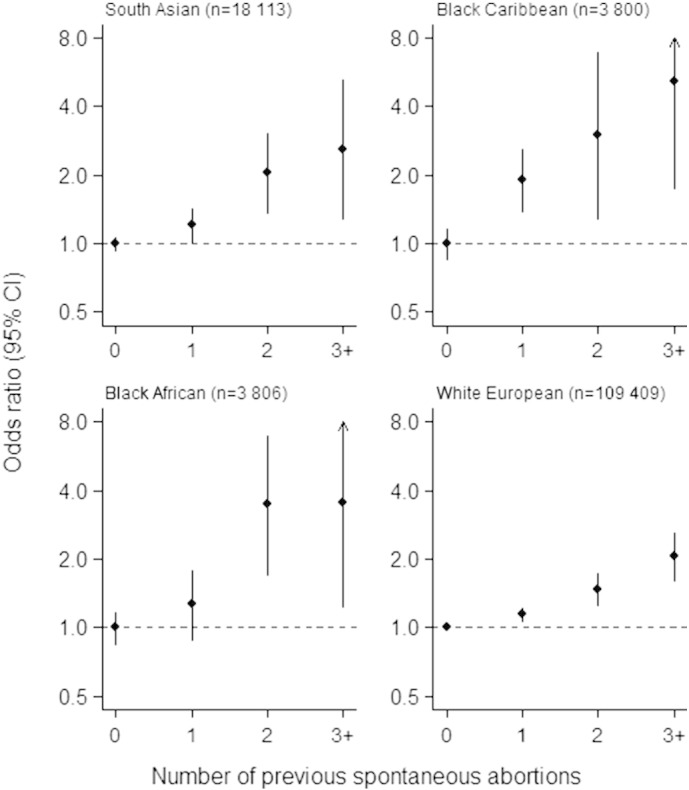
Odds ratios for preterm birth by increasing numbers of reported previous spontaneous abortions, stratified by maternal racial group and using non-imputed data (n = 135 130). Abbreviation: CI, confidence interval.

Positive associations between previous spontaneous abortion and preterm birth were found for all races (Table 4). After adjustment for maternal characteristics, the strongest association found was for black African women. A greater number of previous spontaneous abortions was associated with greater odds of preterm birth for all races (*P* < 0.004 for all, Wald test). This increase was most pronounced in black African and black Caribbean women, for whom three or more spontaneous abortions was associated with an approximately four-fold increase in preterm birth incidence (Fig. 1).

No associations or dose–response relationships were found between previous spontaneous abortion and small-for-gestational-age neonates in any race (Table 4).

## 4 Discussion

Strong, independent relationships between both black African and black Caribbean races and spontaneous abortion relative to white European race have been reported. The factors strongly associated with previous spontaneous abortion were maternal age at delivery, BMI, smoking, and diabetes status. In black African and South Asian women, the size of the association increased with age more rapidly than in white Europeans. The rate of spontaneous abortion also rose with BMI, most strikingly in South Asians. Previous spontaneous abortion was associated with preterm birth, most prominently in black African women, and significant dose–response relationships were found in all racial groups. Previous spontaneous abortion was not associated with low birth weight for any racial group.

A few previous studies have also found a greater incidence of spontaneous abortion in black African and Afro-Caribbean women [3,4]. The association between spontaneous abortion and subsequent preterm birth has also been found previously [5], although a stronger association in black African women relative to white Europeans does not seem to have been identified previously. The present findings could reflect differences in healthcare access. Health care is free in the UK, but ethnic minority groups are more likely to access maternity services late in pregnancy and less likely to have a scan at 20 weeks than are their white British counterparts [15].

Health problems which might underlie the present findings include higher rates of uterine fibroids [16] and systemic lupus erythematosus [17] in black women—disorders that are known risk factors for spontaneous abortion and preterm birth [18,19]—and higher rates of polycystic ovarian syndrome in South Asian women. Symptoms of polycystic ovarian syndrome present at a younger age in South Asian women than in white women [20]. The disorder causes reproductive problems—42%–73% of affected women [21] experience spontaneous abortions—and it is associated with a greater BMI—approximately 50% of patients are overweight or obese, with menstrual irregularities occurring more frequently in obese than normal-weight women [22].

Additionally, it has been proposed that surgical management of spontaneous abortion without cervical pretreatment could mediate the association between spontaneous abortion and preterm birth by weakening the cervix, leading to preterm birth in the next continuing pregnancy [23]. Although management of spontaneous abortion is unlikely to vary by race, there are racial disparities in cervical insufficiency. Black women are at two-fold greater risk of cervical insufficiency than white women [24], which could compound any cervical damage arising from the management of spontaneous abortion.

Data on the number of previous spontaneous abortions used in the present study were collected prospectively, avoiding any bias toward a particular hypothesis regarding the pregnancy’s outcome. There is a risk of under-reporting, but this is unlikely to vary by race, and the rates of spontaneous abortion reported are similar to other populations in the UK and other high-income countries [3,5]. The North West Thames dataset has been extensively validated [25,26] and a substantial number of variables collected, allowing for appropriate adjustment and assessment of interaction. Data regarding confounders and effect modifiers were collected after spontaneous abortion, which might have led to residual confounding, although many of variables reflect long-term health outcomes that are unlikely to change (e.g. history of epilepsy and renal disease). Additionally, the interpregnancy interval between an initial spontaneous abortion and subsequent pregnancy is, on average, less than 6 months [27], so any residual confounding will be minimal and is unlikely to vary by race. Finally, because only women in their first pregnancy were included, results are only generalizable to nulliparous women.

In summary, black Caribbean and black African nulliparous women had an increased incidence of spontaneous abortion, and the effect of spontaneous abortions on subsequent preterm birth varied by racial group. In South Asian women, incidence of spontaneous abortion increased with age and BMI, which could relate to higher rates of polycystic ovary syndrome—yet another reason to encourage South Asian women to avoid being overweight.

The influence of age on spontaneous abortion varied by race, which could be important for counseling in relation to the timing of pregnancy, whereas the differential effect of spontaneous abortion on preterm birth by race has implications for clinical practice. Current practice involves identifying women who could be at increased risk of preterm birth, and providing regular assessment with a view to optimizing outcomes for preterm newborns. The present findings contribute to the identification of at-risk women. Overall, the findings should prompt detailed studies of the factors known to affect rates of spontaneous abortion in relation to race.

## Figures and Tables

**Table 1:** Maternal characteristics, reported previous spontaneous abortions, and pregnancy outcomes, by maternal racial group.^a^

	White European (n = 158 587)	Black African (n = 6659)	Black Caribbean (n = 5808)	South Asian (n = 24 986)	Total (n = 196 040)	*P* value^b^
Spontaneous abortions						< 0.001
0	136 727 (86.2)	5659 (85.0)	4897 (84.3)	22 049 (88.2)	169 332 (86.4)	
1	17 744 (11.2)	768 (11.5)	715 (12.3)	2431 (9.7)	21 658 (11.0)	
2	2968 (1.9)	155 (2.3)	139 (2.4)	362 (1.4)	3624 (1.8)	
≥ 3	1148 (0.7)	77 (1.2)	57 (1.0)	144 (0.6)	1426 (0.7)	
Maternal age at first delivery, y	27 (2–31)	26 (23–29)	25 (21–29)	25 (22–29)	27 (23–30)	< 0.001
BMI^c^	23 (21–26)	24 (21–27)	24 (21–27)	22 (20–25)	23 (21–26)	< 0.001
Marital status^d^						< 0.001
Married	97 697 (65.4)	3412 (55.5)	1559 (28.9)	23 006 (94.6)	125 674 (67.8)	
Unmarried with partner	27 188 (18.2)	1407 (22.9)	1532 (28.4)	869 (3.6)	30 996 (16.7)	
Unmarried without partner	24 536 (16.4)	1329 (21.6)	2303 (42.7)	431 (1.8)	28 599 (15.4)	
History of hypertension^e^						< 0.001
No	154 906 (98.3)	6503 (98.5)	5629 (97.6)	24 739 (99.4)	191 777 (98.4)	
Yes	2733 (1.7)	101 (1.5)	136 (2.4)	143 (0.6)	3113 (1.6)	
History of epilepsy^f^						< 0.001
No	156 912 (99.4)	6592 (99.7)	5752 (99.6)	24 795 (99.6)	194 051 (99.4)	
Yes	971 (0.6)	17 (0.3)	23 (0.4)	95 (0.4)	1106 (0.6)	
History of renal disease^g^						< 0.001
No	157 141 (99.6)	6600 (99.7)	5744 (99.7)	24 858 (99.8)	194 343 (99.6)	
Yes	674 (0.4)	17 (0.3)	19 (0.3)	55 (0.2)	765 (0.4)	
History of induced abortion						< 0.001
No	134 375 (84.7)	4789 (71.9)	4393 (75.6)	23 202 (92.9)	166 759 (85.1)	
Yes	24 212 (15.3)	1870 (28.1)	1415 (24.4)	1784 (7.1)	29 281 (14.9)	
History of cardiac disease^h^						< 0.001
No	156 330 (99.2)	6570 (99.5)	5705 (99.1)	24 781 (99.5)	193 386 (99.2)	
Yes	1279 (0.8)	35 (0.5)	52 (0.9)	119 (0.5)	1485 (0.8)	
Diabetes status^i^						< 0.001
No diabetes	156 333 (99.0)	6493 (98.2)	5664 (98.3)	24 365 (97.8)	192 855 (98.8)	
Gestational diabetes	1032 (0.7)	109 (1.7)	77 (1.3)	504 (2.1)	1722 (0.9)	
Established diabetes	516 (0.3)	11 (0.2)	21 (0.3)	52 (0.2)	600 (0.3)	
Smoking status^j^						< 0.001
Non-smoker	121 900 (78.1)	6158 (94.3)	4636 (81.1)	24 012 (97.9)	156 706 (81.3)	
Current smoker	34 135 (21.9)	370 (5.7)	1078 (18.9)	519 (2.1)	36 102 (18.7)	
Carstairs deprivation score^k^						< 0.001
1 (least deprived)	34 423 (23.9)	337 (6.0)	276 (5.5)	2125 (9.1)	37 161 (20.9)	
2	35 424 (24.6)	629 (11.1)	483 (9.5)	2939 (12.6)	39 475 (22.1)	
3	36 185 (25.1)	1136 (20.1)	953 (18.8)	5462 (23.4)	43 736 (24.5)	
4	26 204 (18.2)	1616 (28.6)	1483 (29.3)	6366 (27.3)	35 669 (20.0)	
5 (most deprived)	11 921 (8.3)	1934 (34.2)	1869 (36.9)	6456 (27.7)	22 180 (12.4)	
Year of delivery						< 0.001
1988–1990	39 360 (24.8)	1230 (18.5)	1409 (24.3)	6036 (24.2)	48 035 (24.5)	
1991–1993	39 531 (24.9)	1694 (25.4)	1565 (26.9)	6552 (26.2)	49 342 (25.2)	
1994–1996	33 723 (21.3)	1511 (22.7)	1210 (20.8)	4930 (19.7)	41 374 (21.1)	
1997–2000	45 973 (29.0)	2224 (33.4)	1624 (28.0)	7468 (29.9)	57 289 (29.2)	
Gestational age at birth^l^						< 0.001
Term birth (≥ 37 wk)	136 025 (93.4)	5519 (89.9)	4741 (88.7)	21 086 (91.8)	167 371 (92.9)	
Preterm birth (< 37 wk)	9618 (6.6)	619 (10.1)	603 (11.3)	1876 (8.2)	12 716 (7.1)	
Birth weight^l^						< 0.001
Not in lowest 10%	135 005 (92.7)	5475 (89.2)	4740 (88.7)	18 345 (79.9)	163 565 (90.8)	
In lowest 10%	10 638 (7.3)	663 (10.8)	604 (11.3)	4617 (20.1)	16 522 (9.2)	

Abbreviation: BMI, body mass index (calculated as weight in kilograms divided by the square of height in meters).

a Values are given as number (percentage) or median (interquartile range), unless indicated otherwise.

b χ^2^ test for categorical variables and Kruskal-Wallis test for continuous variables.

c n = 161 594 (white European n = 131 560; black African n = 5027; black Caribbean n = 4683; South Asian n = 20 324).

d n = 185 269 (white European n = 149 421; black African n = 6148; black Caribbean n = 5394; South Asian n = 24 306).

e n = 194 890 (white European n = 157 639; black African n = 6604; black Caribbean n = 5765; South Asian n = 24 882).

f n = 195 157 (white European n = 157 883; black African n = 6609; black Caribbean n = 5775; South Asian n = 24 890).

g n = 195 108 (white European n = 157 815; black African n = 6617; black Caribbean n = 5763; South Asian n = 24 913).

h n = 194 871 (white European n = 157 609; black African n = 6605; black Caribbean n = 5757; South Asian n = 24 900).

i n = 195 177 (white European n = 157 881; black African n = 6613; black Caribbean n = 5762; South Asian n = 24 921).

j n = 192 808 (white European n = 156 035; black African n = 6528; black Caribbean n = 5714; South Asian n = 24 531).

k n = 178 221 (white European n = 144 157; black African n = 5652; black Caribbean n = 5064; South Asian n = 23 348).

l n = 180 087 (white European n = 145 643; black African n = 6138; black Caribbean n = 5344; South Asian n = 22 962).

**Table 2:** Independent factors associated with reported previous spontaneous abortion, by maternal racial group and using imputed data (n = 196 040).a,b

	White European (n = 158 587)	Black African (n = 6659)	Black Caribbean (n = 5808)	South Asian (n = 24 986)	Total (n = 196 040)
Maternal age at delivery	1.06 (1.06–1.06)^c^	1.09 (1.07–1.10)^c^	1.07 (1.06–1.09)^c^	1.08 (1.07–1.09)^c^	1.06 (1.06–1.07)^c^
BMI	1.02 (1.02–1.02)^c^	1.03 (1.01–1.05)^c^	0.99 (0.98–1.01)	1.04 (1.03–1.05)^c^	1.02 (1.02–1.03)^c^
Marital status^d^					
Unmarried with partner	0.93 (0.89–0.97)^c^	1.01 (0.83–1.23)	1.04 (0.85–1.28)	0.88 (0.71–1.11)	0.94 (0.91–0.98)^c^
Unmarried without partner	0.90 (0.86–0.95)^c^	0.97 (0.80–1.17)	0.88 (0.72–1.07)	0.86 (0.62–1.20)	0.91 (0.87–0.95)^c^
History of hypertension	1.00 (0.90–1.11)	0.80 (0.46–1.40)	1.09 (0.70–1.68)	1.32 (0.85–2.04)	1.01 (0.91–1.11)
History of epilepsy	1.17 (0.98–1.40)	0.96 (0.26–3.52)	0.29 (0.04–2.16)	0.85 (0.44–1.65)	1.12 (0.95–1.33)
History of renal disease	1.14 (0.92–1.40)	2.02 (0.67–6.08)	0.79 (0.22–2.82)	0.52 (0.19–1.46)	1.10 (0.90–1.34)
History of induced abortion	0.94 (0.91–0.98)^c^	0.82 (0.70–0.96)^c^	0.90 (0.76–1.07)	1.06 (0.92–1.23)	0.94 (0.91–0.98)^c^
History of cardiac disease	1.21 (1.04–1.40)^c^	1.42 (0.59–3.39)	1.81 (0.95–3.46)	0.99 (0.56–1.74)	1.22 (1.06–1.40)^c^
Diabetes status^e^					
Established diabetes	1.31 (1.04–1.65)^c^	3.83 (1.07–13.70)^c^	2.08 (0.77–5.56)	0.99 (0.46–2.13)	1.35 (1.10–1.67)^c^
Gestational diabetes	1.25 (1.07–1.46)^c^	1.19 (0.75–1.90)	2.09 (1.27–3.44)^c^	1.49 (1.20–1.87)^c^	1.38 (1.22–1.56)^c^
Smoking^f^	1.36 (1.31–1.41)^c^	0.96 (0.70–1.34)	1.50 (1.26–1.82)^c^	1.75 (1.38–2.22)^c^	1.37 (1.33–1.42)^c^
Carstairs deprivation score^g^					
2	0.97 (0.93–1.01)	1.06 (0.75–1.48)	1.14 (0.77–1.68)	1.08 (0.90–1.28)	0.98 (0.94–1.02)
3	0.99 (0.95–1.04)	0.89 (0.63–1.26)	0.92 (0.63–1.35)	1.07 (0.92–1.25)	1.00 (0.96–1.04)
4	1.04 (1.00–1.09)	1.00 (0.73–1.36)	1.00 (0.70–1.42)	1.09 (0.93–1.28)	1.04 (1.00–1.09)
5	1.07 (1.01–1.14)^c^	0.91 (0.66–1.25)	1.15 (0.82–1.62)	1.13 (0.97–1.33)	1.06 (1.01–1.12)^c^
Year of delivery	1.01 (1.01–1.01)^c^	0.99 (0.98–1.01)	1.01 (0.99–1.03)	1.01 (1.00–1.02)	1.01 (1.01–1.01)^c^
Racial group^h^					
Black African	–	–	–	–	1.20 (1.12–1.29)^c^
Black Caribbean	–	–	–	–	1.31 (1.21–1.41)^c^
South Asian	–	–	–	–	0.95 (0.91–1.00)^c^

Abbreviation: BMI, body mass index (calculated as the weight in kilograms divided by the square of height in meters).

a Values are given as adjusted odds ratio (95% confidence interval).

b Adjusted for diabetes status, marital status, smoking status, year of delivery, maternal age at delivery, Carstairs deprivation score, BMI, history of induced abortion, hypertension, renal disease, cardiac disease, and epilepsy.

c *P* < 0.05.

d Reference group: married.

e Reference group: no diabetes.

f Reference group: non-smoker.

g Reference group: 1 (least deprived).

h Reference group: white European.

**Table 3:** Adjusted odds ratios for previous spontaneous abortion according to advancing maternal age and increasing body mass index, by maternal racial group and using non-imputed data (n = 136 208).a,b

	White European (n = 110 447)	Black African (n = 3875)	Black Caribbean (n = 3721)	South Asian (n = 18 165)
BMI				
Underweight	1.01 (0.97–1.05)	0.89 (0.74–1.08)	1.19 (0.98–1.46)	0.77 (0.72–0.82)^c^
Normal	1	1.12 (1.07–1.18)^c^	1.41 (1.35–1.48)^c^	0.93 (0.91–0.96)^c^
Overweight/obese	1.10 (1.09–1.12)^c^	1.45 (1.39–1.51)^c^	1.40 (1.34–1.46)^c^	1.12 (1.09–1.15)^c^
Age, y				
< 20	0.73 (0.71–0.75)^c^	0.55 (0.48–0.62)^c^	0.69 (0.64–0.75)^c^	0.52 (0.49–0.55)^c^
21–25	1	1.02 (0.96–1.08)	1.21 (1.14–1.29)^c^	0.81 (0.78–0.84)^c^
26–30	1.12 (1.10–1.14)^c^	1.38 (1.32–1.45)^c^	1.55 (1.47–1.64)^c^	1.09 (1.05–1.12)^c^
31–35	1.50 (1.47–1.53)^c^	2.25 (2.10–2.40)^c^	2.09 (1.95–2.25)^c^	1.62 (1.55–1.69)^c^
≥ 35	2.42 (2.36–2.48)^c^	2.25 (1.98–2.56)^c^	3.07 (2.72–3.45)^c^	2.70 (2.53–2.90)^c^

Abbreviation: BMI, body mass index (calculated as weight in kilograms divided by the square of height in meters).

a Values are given as adjusted odds ratio (95% confidence interval).

b Adjusted for diabetes status, marital status, smoking status, year of delivery, maternal age at delivery, Carstairs deprivation score, BMI, history of induced abortion, hypertension, renal disease, cardiac disease, and epilepsy.

c *P* < 0.001

**Table 4:** Odds of adverse obstetric outcomes after spontaneous abortion, by maternal racial group and using imputed data (n = 180 087).

Outcome	Unadjusted odds ratios (95% CI)				Adjusted odds ratios (95% CI)^a^			
	White European (n = 145 643)	Black African (n = 6138)	Black Caribbean (n = 5344)	South Asian (n = 22 962)	White European (n = 145 643)	Black African (n = 6138)	Black Caribbean (n = 5344)	South Asian (n = 22 962)
Preterm birth	1.21 (1.17–1.26)	1.53 (1.35–1.73)	1.25 (1.08–1.45)	1.25 (1.08–1.45)	1.21 (1.16–1.26)	1.47 (1.29–1.67)	1.24 (1.07–1.44)	1.22 (1.18–1.27)
Small-for-gestational-age birth^b^	1.01 (0.97–1.04)	0.90 (0.77–1.04)	0.89 (0.75–1.04)	0.98 (0.91–1.05)	0.99 (0.95–1.03)	0.91 (0.78–1.07)	0.88 (0.74–1.04)	1.02 (0.95–1.10)

Abbreviation: CI, confidence interval.

a Adjusted for diabetes status, marital status, smoking status, year of delivery, maternal age at delivery, Carstairs deprivation score, body mass index, history of induced abortion, hypertension, renal disease, cardiac disease, and epilepsy.

b Lowest 10th percentile, corrected for sex and gestational age at birth.
